# Dynamic state of plasmid genomic architectures resulting from XerC/D-mediated site-specific recombination in *Acinetobacter baumannii* Rep_3 superfamily resistance plasmids carrying *bla_OXA-58_*- and Tn*aphA6*-resistance modules

**DOI:** 10.3389/fmicb.2023.1057608

**Published:** 2023-02-09

**Authors:** Lucía Giacone, M. Marcela Cameranesi, Rocío I. Sanchez, Adriana S. Limansky, Jorgelina Morán-Barrio, Alejandro M. Viale

**Affiliations:** Departamento de Microbiología, Facultad de Ciencias Bioquímicas y Farmacéuticas, CONICET, Instituto de Biología Molecular y Celular de Rosario (IBR), Universidad Nacional de Rosario (UNR), Rosario, Argentina

**Keywords:** *Acinetobacter baumannii*, carbapenem resistance, resistance plasmids, OXA-58 carbapenemase, XerC/D site-specific recombination, multiple pXerC/D sites, plasmid shuffling, plasmid dynamics and evolution

## Abstract

The acquisition of *bla*_OXA_ genes encoding different carbapenem-hydrolyzing class-D β-lactamases (CHDL) represents a main determinant of carbapenem resistance in the nosocomial pathogen *Acinetobacter baumannii*. The *bla_OXA-58_* gene, in particular, is generally embedded in similar resistance modules (RM) carried by plasmids unique to the *Acinetobacter* genus lacking self-transferability. The ample variations in the immediate genomic contexts in which *bla_OXA-58_*-containing RMs are inserted among these plasmids, and the almost invariable presence at their borders of non-identical 28-bp sequences potentially recognized by the host XerC and XerD tyrosine recombinases (pXerC/D-like sites), suggested an involvement of these sites in the lateral mobilization of the gene structures they encircle. However, whether and how these pXerC/D sites participate in this process is only beginning to be understood. Here, we used a series of experimental approaches to analyze the contribution of pXerC/D-mediated site-specific recombination to the generation of structural diversity between resistance plasmids carrying pXerC/D-bounded *bla*_OXA-58_- and Tn*aphA6*-containing RM harbored by two phylogenetically- and epidemiologically-closely related *A. baumannii* strains of our collection, Ab242 and Ab825, during adaptation to the hospital environment. Our analysis disclosed the existence of different *bona fide* pairs of recombinationally-active pXerC/D sites in these plasmids, some mediating reversible intramolecular inversions and others reversible plasmid fusions/resolutions. All of the identified recombinationally-active pairs shared identical GGTGTA sequences at the cr spacer separating the XerC- and XerD-binding regions. The fusion of two Ab825 plasmids mediated by a pair of recombinationally-active pXerC/D sites displaying sequence differences at the cr spacer could be inferred on the basis of sequence comparison analysis, but no evidence of reversibility could be obtained in this case. The reversible plasmid genome rearrangements mediated by recombinationally-active pairs of pXerC/D sites reported here probably represents an ancient mechanism of generating structural diversity in the *Acinetobacter* plasmid pool. This recursive process could facilitate a rapid adaptation of an eventual bacterial host to changing environments, and has certainly contributed to the evolution of *Acinetobacter* plasmids and the capture and dissemination of *bla*_OXA-58_ genes among *Acinetobacter* and non-*Acinetobacter* populations co-residing in the hospital niche.

## Introduction

The main cause of carbapenem resistance among multidrug-resistant (MDR) clinical strains of the opportunistic pathogen *Acinetobacter baumannii* and its phylogenetically-related species of the *A. calcoaceticus*-*A. baumannii* (ACB) complex is represented by acquired *bla*_OXA_ genes encoding carbapenem-hydrolyzing class-D β-lactamases (CHDL) of the OXA-23, OXA-40/24, OXA-58, OXA-143, and OXA-235 groups (Roca et al., 2012; Carattoli, 2013; Evans and Amyes, 2014; Zander et al., 2014; Da Silva and Domingues, 2016; Hamidian and Nigro, 2019). The *bla*_OXA-58_ gene, in particular, is generally found in iteron plasmids endowed with replication initiation protein genes (*repAci*) encoding members of the Rep_3 (PF01051) superfamily (Rep_3 plasmids) unique to the *Acinetobacter* genus (Zarrilli et al., 2008; Roca et al., 2012; Carattoli, 2013; Evans and Amyes, 2014; Fu et al., 2014; Zander et al., 2014; Da Silva and Domingues, 2016; Cameranesi et al., 2017, 2018; Lean and Yeo, 2017; Salto et al., 2018; Hamidian and Nigro, 2019; Wang et al., 2020; Hamidian et al., 2021; Liu et al., 2021; Brito et al., 2022; Jones et al., 2022). A number of remarkable features distinguish the *bla*_OXA-58_-containing structures carried by these plasmids, including the different genetic contexts in which they are inserted and the almost invariable presence at their borders of a variable number of short sequences displaying homology to the 28-bp chromosomal *dif* site recognized by the XerC and XerD tyrosine recombinases which have been alternatively designated Re27, p*dif*, or pXerC/D sites. This has led to suggestions that these pXerC/D-bounded structures could constitute modules endowed with horizontal mobilization abilities, but how these pXerC/D sites participate in the lateral mobilization of the structures they encircle is only beginning to be understood (Cameranesi et al., 2018).

The XerC/D SSR system, ubiquitous among bacteria, serves primary physiological roles in the separation of circular chromosomes prior to cell division by resolving dimers generated by homologous recombination during DNA replication (Cornet et al., 1994; Colloms et al., 1996; Rajeev et al., 2009; Crozat et al., 2014; Ramirez et al., 2014; Balalovski and Grainge, 2020). This system is unique among other SSR systems in that it employs two homologous tyrosine recombinases, XerC and XerD, which recognize a core 28-bp sequence known as *dif* organized in an imperfect palindrome composed of 11-bp each XerC- and XerD-binding regions, separated by a central spacer region (cr) of 6 bp where recombination occurs. Some ColE1-type plasmids of the *Enterobacteriaceae* use the XerC/D SSR system of their hosts to resolve their own multimeric forms, thus avoiding segregational instability. *Acinetobacter* plasmids, on the contrary, are unique in the sense that they contain not a single but several pXerC/D-like sites not necessarily located in direct orientations, which even display some sequence differences between them (Cameranesi et al., 2018). These features suggested functions other than participating only in the resolution of multimers and, in this context, sequence comparison analyses of a number of *Acinetobacter* plasmids provided evidence of their participation in DNA inversions and fusions/resolutions (Cameranesi et al., 2018; Balalovski and Grainge, 2020; Wang et al., 2020; Alattraqchi et al., 2021; Hamidian et al., 2021; Jones et al., 2022). Yet, experimental evidence supporting their role in SSR events is still scarce, and many questions remain including whether all the pXerC/D sites inferred in *Acinetobacter* plasmids are active in recombination, the kind of exchanges they can mediate, the factors that govern these reactions, and their effects on the evolutionary dynamics of the plasmids in which they are embedded and the hosts that eventually carry them.

We previously reported first empirical evidences indicating the existence of a recombinationally-active pair mediating the fusion of two plasmids into a co-integrate in *A. baumannii* cells of our collection (Cameranesi et al., 2018). Moreover, we observed that this co-integrate could also resolve in these cells, following an intramolecular SSR event now mediated by the pair of hybrid pXerC/D sites resulting from the previous intermolecular fusion. More recently, reconstructions of the evolutionary history of the *bla*_OXA-58_-containing *A. baumannii* plasmid pA388 (Jones et al., 2022) also showed the existence of intramolecular inversions mediated by recombinationally active pairs of pXerC/D sites. The overall observations indicated that at least some of the pXerC/D sites inferred in *Acinetobacter* plasmids can constitute *bona fide* recombinationally active pairs, supporting roles in the evolution of the plasmid molecules in which they are embedded.

We recently characterized by whole genome sequencing (WGS) a number of phylogenetically- and epidemiologically-related MDR *A. baumannii* strains belonging to the clonal complex CC15 (Pasteur scheme) prevalent in South America (Matos et al., 2019; Cameranesi et al., 2020; Brito et al., 2022). Comparative genome analysis among two carbapenem-resistant strains of this collection, Ab242 and Ab825, indicated extensive chromosomal synteny between them but also disclosed a much larger number of acquired mobile elements in the Ab825 genome resulting in the inactivation of many exposed cell structures, including protective systems against external aggressors, effectors of the innate immune system, and outer membrane proteins related to carbapenem resistance and virulence, in a process most probably associated to the adaptation of this strain to the hospital environment (Mussi et al., 2005; Cameranesi et al., 2020; Labrador-Herrera et al., 2020). Assembly of the predominant plasmid forms based on WGS data indicated that Ab825 and Ab242 harbored plasmids endowed with almost identical *bla*_OXA-58_- and Tn*aphA6*-containing resistance modules (RMs) bounded with pXerC/D sites, but disclosed differences between strains in the structural organization of these resistance plasmids.

We sought to analyze in this work whether and how pXerC/D-mediated recombination contributed to the generation of structural diversity between Ab242 and Ab825 plasmids. By developing a series of experimental approaches (see Supplementary Figure S2 for details), we disclosed the existence of *bona fide* pairs of recombinationally-active pXerC/D sites among them, some mediating reversible intramolecular inversions and others reversible plasmid fusions/resolutions. This reversible remodeling of *Acinetobacter* plasmid structures mediated by different pairs of pXerC/D sites significantly impacts the possibilities of adaptation of an eventual host to varying and/or challenging environments, the evolutionary dynamics of the plasmids involved in this process, and the consequent dissemination of *bla*_OXA-58_ genes and other adaptive determinants among the bacterial community.

## Materials and methods

### Bacterial strains and growth conditions

The MDR, carbapenem-resistant *A. baumannii* clinical strains Ab242 and Ab825 were isolated from hospitalized patients in a public healthcare center (HECA) of Rosario, Argentina. These two strains were assigned to the clonal complex CC15 (Pasteur MLST scheme), and were characterized recently by WGS and comparative genome sequence analysis (Cameranesi et al., 2020).

The *A. nosocomialis* strain M2 (AnM2) is an antimicrobial susceptible ACB strain lacking indigenous plasmids (Carruthers et al., 2013), and was used as recipient for electrotransformation assays with plasmid mixtures extracted from the *A. baumannii* clinical strains analyzed (see below, and also Supplementary Figure S2). Similarly, the collection *A. baumannii* ATCC17978 strain (Ab17978) was also used for the same purpose.

All bacterial cells were grown in Lysogeny Broth (LB) liquid medium at 37°C under aerobic conditions with vigorous shaking, or in LB solid medium prepared by supplementing the LB liquid medium with 1.5% Difco Agar. When necessary, culture media were supplemented with the antimicrobials indicated at the concentrations stated in the text or in the legends to figures.

### Growth of clonal populations of *Acinetobacter* strains and plasmid isolation

We used the general scheme described previously (Cameranesi et al., 2018). In short, isolated colonies of the clinical strains Ab242 or Ab825, or the selected IPM-resistant AnM2 or Ab17978 strains generated in this work, were independently obtained after streaking frozen stocks of the corresponding cells on LB agar media supplemented with 2 μg/ml IPM, followed by incubation at 37°C overnight. Then, clonal populations of each of these strains were generated by inoculating 10 ml of liquid LB medium supplemented with 2 μg/ml IPM with cells picked up from an isolated colony, followed by incubation at 37°C under gentle aeration until confluence. Plasmid extraction was performed from these cultures using the Wizard® Plus SV Minipreps DNA Purification Systems (Promega, WI, USA) and their quality was routinely analyzed by 0.7% agarose gel electrophoresis/ethidium bromide staining.

### PCR identification of recombination events mediated by active sister pairs of pXerC/D sites in *Acinetobacter* plasmids

The plasmid mixtures obtained from the clonal cultures of the different *Acinetobacter* strains were independently used as templates in PCR assays aimed to detect recombination events mediated by particular pairs of pXerC/D sites (Supplementary Figures S1, S2). The primers employed were used in adequate combinations to detect the different pXerC/D sites and corresponding genetic contexts (see Supplementary Table S1). Primer pair H was additionally employed to detect the presence of plasmids containing the *bla*_OXA-58_ carbapenemase gene in isolated colonies of the Ab242 or Ab825 strains, as well as in IPM-resistant AnM2 or Ab17978 colonies obtained after transformation with Ab242 or Ab825 plasmids (see below). The amplification mixtures were analyzed by agarose gel electrophoresis, and the obtained amplicons were subsequently cloned into pGEM-T Easy (Promega) for further sequence analysis. Plasmids containing cloned DNA were analyzed by restriction mapping, and selected inserts were sequenced and subjected to comparative sequence analysis with the different predominant plasmid structures assembled from WGS data in the Ab242 or Ab825 strains to identify recombination events mediated by active pairs of pXerC/D sites (see Supplementary Figure S2 for details).

### Transformation of AnM2 and *Acinetobacter baumannii* Ab17978 cells

Plasmid mixtures extracted from clonal cultures of the Ab242 or Ab825 strains were used for transformation assays employing electrocompetent AnM2 or Ab17978 cells as receptors, following previously described procedures (Ravasi et al., 2011). The rationale here was to obtain separate clonal lineages, in which each lineage originated from an individual cell initially transformed with a single plasmid structural variant carrying the RM from those present in the plasmid mixture (Supplementary Figure S2D). The transformed cells were plated on LB agar supplemented with 2 μg/ml IPM, and incubated 24–48 h at 37°C. Different IPM-resistant AnM2 or Ab17978 colonies developing during this period were analyzed by PCR for the presence of plasmids bearing the *bla*_OXA-58_ gene. Stocks of AnM2 or Ab17978 pure cultures generated from cells testing positive for *bla*_OXA-58_ were stored at −80° C and routinely used to regenerate the independent cultures (clonal populations) used in this work. Plasmids extracted from these clonal cultures were used in turn as templates in PCR reactions aimed to detect structural variants resulting from recombination events mediated by particular sister pairs of pXerC/D sites.

### DNA sequencing, plasmid assembly, and comparative sequence analyses

The assembly of Ab242 plasmids was described previously (Cameranesi et al., 2018). Ab825 genomic DNA was isolated using the DNeasy Blood and Tissue kit (Qiagen), and its genomic sequence was obtained using a paired-end strategy using an Illumina MiSeq sequencer at the National Institute of Health (Lisbon, Portugal). Reads were subjected to quality assessment and further assembly using Velvet version 1.2.10. The *de novo* assembly process was optimized using the Velvet Optimiser script version 2.2.5. Gaps remaining in contigs corresponding to Ab825 plasmid sequences were closed using PCR with specifically designed primers employing Ab825 plasmid extracts as templates (Supplementary Figure S3).

The Rapid Annotation using Subsystem Technology standard operating procedures (RAST; http://rast.nmpdr.org/seedviewer.cgi; Aziz et al., 2008) and the National Center for Biotechnology Information database (NCBI, U.S. National Library of Medicine, Bethesda MD, USA) were used to annotate the open reading frames (ORFs) found in the different plasmids. Insertion sequence (IS) elements were detected using IS Finder (Siguier et al., 2006; https://www-is.biotoul.fr/) and ISSaga (Varani et al., 2011). Plasmid pXerC/D-like core sequences were detected as described previously (Cameranesi et al., 2018).

The DNA sequences of the PCR fragments cloned into pGem-T were conducted at the Sequencing Facilities of Maine University, Orono (ME, U.S.A.) or at Macrogen (Seoul, Korea).

## Results

### Comparative analysis of *Acinetobacter baumannii* Ab242 and Ab825 carbapenem resistance plasmids

Detailed comparative sequence analysis of sequence-related plasmids found in different *A. baumannii* strains represents a useful tool to detect the recombination mechanism (s) that could account for any observed structural heterogeneity (Cameranesi et al., 2018; Wang et al., 2020; Alattraqchi et al., 2021; Hamidian et al., 2021; Jones et al., 2022). In the case of SSR events mediated by sister pairs of pXerC/D sites, these comparisons are also pivotal for the design of specific PCR primers hybridizing at the immediate neighboring regions associated to each of the pXerC/D-like half-sites that provided for the sister pair (Supplementary Figure S1). PCR analysis using appropriate combinations of primers and as template a plasmid mixture extracted from a clonal population of the studied strain (s), followed by sequence analysis of the obtained amplicons, could then verify whether plasmid structural variants (PSVs) carrying the inferred pXerC/D-mediated inversions and/or fusions are simultaneously present in the analyzed plasmid population (Supplementary Figure S2). This coexistence, in turn, signals the possible operation of a dynamic interconversion state of PSVs resulting from pXerC/D-mediated reversible SSR events in the analyzed cell populations.

**Figure 1:**
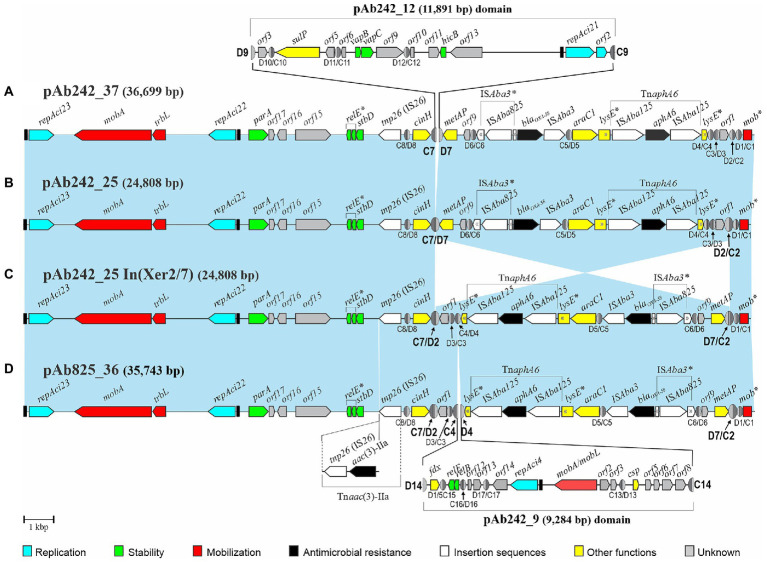
Comparisons of the genetic structures of four plasmid structural variants carrying identical *bla*_OXA-58_- and Tn*aphA6*-containing resistance modules bordered by multiple pXerC/D sites identified among related *Acinetobacter baumannii* clinical strains studied in this work. Linear representations of four Rep_3-based multi-replicon resistance plasmids characterized in the Ab242 and Ab825 strains. For comparison purposes all plasmids were drawn starting from the iteron sequences region located immediately upstream of the *repAci23* replication initiation protein gene (black rectangle at the left), with the orientations and extents of the different genes and open reading frames also shown. The genes containing premature termination codons, insertions, and pseudogenes are labeled with asterisks. The genes encoding the different replication initiation proteins belonging to the Rep_3 superfamily are denoted as *repAci* followed by their assigned denominations, with their preceding iteron sequences indicated as black rectangles. The carbapenem- and aminoglycoside-resistance module (RM) encompassing the *bla*_OXA-58_- and *aphA6* resistant genes and accompanying elements is found in all plasmids associated to multiple pXerC/D sites located in different orientations. The light-blue shaded background interconnecting the structures of the different plasmids denote homologous regions displaying nucleotide sequence identity ≥99%, the cross sectors indicate the inverted regions found between plasmids. Antimicrobial resistance genes are labeled in black and IS elements in white, with their corresponding denominations indicated above each plasmid structure. The extension of the IS*Aba125*-based composite transposon Tn*aphA6* inserted within the *lysE* gene is also indicated. The different pXerC/D-like sites inferred in these plasmids are shown as ovals (not drawn to scale), with the XerC (C) and XerD (D) recognition sequences depicted as dark and light gray semi-ovals, respectively. Their designations (see Cameranesi et al., 2018 for numbering and sequence details) and corresponding polarities are indicated below the structures. The pXerC/D sites identified as involved in SSR have been enlarged, and their designations highlighted in bold letters. **(A)** pAb242_37 structure. This tri-replicon resulted from an intermolecular fusion mediated by the pXerC/D pair C7/D7 and C9/D9 sites located in pAb242_25 and pAb242_12, respectively (Cameranesi et al., 2018). The structure of pAb242_12 and its insertion site into pAb242_25 are indicated above the structure, and resulted in the generation of a directly-oriented pair of (hybrid) sites formed by C7/D9 and C9/D7. **(B)** pAb242_25. This bi-replicon was assembled from WGS data analysis obtained from the Ab242 strain. **(C)** pAb242_25 In(Xer2/7). Plasmid structural variant of pAb242_25 described in this work, in which the region located between the oppositely-oriented sites C7/D7 and D2/C2 is inverted. As the result, a novel pair of oppositely-oriented (hybrid) sites, C7/D2 and D7/C2, was generated. **(D)** pAb825_36. Plasmid assembled from WGS data analysis of the Ab825 strain followed by gap closure using PCR with specifically-designated primers (see Supplementary Figure S3). Sequence analysis indicated that this tri-replicon was formed by the fusion of a plasmid much similar in structure to pAb242_25 In(Xer2/7) and another plasmid identical to pAb242_9, mediated by the pair of pXerC/D sites C4/D4 and C14/D14 located in pAb242_25 In(Xer2/7) and pAb242_9, respectively. This fusion resulted in the generation of a directly-oriented pair of (hybrid) sites, C4/D14 and C14/D4. A complete IS*26*-based composite transposon, Tn*aac(3)*-IIa, was also found in pAb825_36 and its location is also indicated below the plasmid structure. GenBank accession numbers: pAb242_25, KY984047; pAb242_12, KY984046; pAb242_9, KY984045, pAb825_36, MG100202.

WGS data analysis followed by gap closure strategies resulted in the assembly of two predominant circular plasmid forms in the Ab825 strain (Supplementary Figure S3). One of these plasmids of 11,891 bp in size was identical to pAb242_12 previously reported in the Ab242 strain (Cameranesi et al., 2018). Assembly of the second plasmid indicated a circular tri-replicon of 35,743 bp which was designated pAb825_36, and which carried a *bla_OXA-58_*- and Tn*aphA6*-containing RM almost identical to that described in pAb242_25 including its associated pXerC/D sites (Figure 1). These two plasmids also showed additional regions of sequence similarity including two of the replication modules, a *mobA*- and *trbL*-containing mobilization region, and an IS*26* element. However, notorious differences were also found between them. First, the inversion of the DNA region that contains the *bla*_OXA-58_- and Tn*aphA6*-containing RM, which was limited in pAb242_25 by the pXerC/D sites C2/D2 and C7/D7 (Figure 1B) and in pAb825_36 by C2/D7 and C7/D2 (Figure 1D). A detailed comparative analysis of the 28-bp core regions of these pXerC/D sites (Table 1) showed identical GGTGTA cr spacers as well as identical XerD-binding half sites for all of them, but some nucleotide differences at the XerC-binding half-sites. These comparisons, the relative orientations of these sites in the corresponding molecules, and the evidence provided by PCR assays (see Table 2; Supplementary Figure S4 below), indicated that C2/D7 and C7/D2 are in fact hybrid products of a SSR event mediated by an active sister pair composed of C2/D2 and C7/D7, and vice versa. Secondly, other main difference between pAb242_25 and pAb825_36 was represented by an extra DNA region of 9,284 bp in the latter plasmid, which is identical in sequence to a linearized form of plasmid pAb242_9. In pAb825_36 this pAb242_9-like region is bordered by two directly-oriented pXerC/D-like sites, namely C4/D14 and C14/D4, which share identical XerC and XerD sequences but differ in the cr region in two out of six nucleotides (Table 1). Detailed sequence comparisons between these plasmids strongly suggested that pAb825_36 resulted from an intermolecular fusion between a plasmid identical to pAb242_9 and a pAb242_25 structural variant, an event mediated by a pXerC/D pair formed by C14/D14 and C4/D4 (Figure 1D). Third, another difference between the carbapenem-resistance plasmid forms found in Ab242 and Ab825 is the presence in pAb825_36 of an IS*26*-bounded composite transposon-like structure carrying an *aac (3)*-IIa aminoglycoside acetyltransferase gene, designated here Tn*aac(3)*-IIa (Figure 1D). Since a single IS*26* copy was found in a similar location in pAb242_25 (Figure 1B), this resistance structure most probably resulted from an IS*26*-mediated recombination event that incorporated, next to this pre-existing IS*26*, a translocatable unit composed of another IS*26* copy (Harmer et al., 2014) carrying in this case a *aac(3)*-IIa-containing DNA fragment.

**Table 1:** pXerC/D core sequences found in the Ab825 and Ab242 plasmids studied in this work.

Plasmid	pXerC/D site^a^	Nucleotide sequence (5′ → 3′)			Relative orientation in the plasmid structure*^b^*
		XerC domain	cr	XerD domain	
pAb825_36	C7/D2	ATTACGTATAA	GGTGTA	TTATGTTAATT	C → D
	C2/D7	ATTTCGCATAA	GGTGTA	TTATGTTAATT	D → C
	C4/D14	ATTTCGTATAA	CAGCCA	TTATGTTAAAT	C → D
	C14/D4	ATTTCGTATAA	CGCCCA	TTATGTTAAAT	C → D
pAb242_25	C2/D2	ATTTCGCATAA	GGTGTA	TTATGTTAATT	D → C
	C7/D7	ATTACGTATAA	GGTGTA	TTATGTTAATT	C → D
	C4/D4	ATTTCGTATAA	CAGCCA	TTATGTTAAAT	D → C
pAb242_25 In(Xer2/7)	C7/D2	ATTACGTATAA	GGTGTA	TTATGTTAATT	C → D
	C2/D7	ATTTCGCATAA	GGTGTA	TTATGTTAATT	D → C
	C4/D4	ATTTCGTATAA	CAGCCA	TTATGTTAAAT	C → D
pAb242_12 (pAb825_12)	C9/D9	ATTTCGTATAA	GGTGTA	TTATGTTATTT	-
pAb242_9	C14/D14	ATTTCGTATAA	CGCCCA	TTATGTTAAAT	-
pAb242_37	C7/D9	ATTACGTATAA	GGTGTA	TTATGTTATTT	C → D
	C9/D7	ATTTCGTATAA	GGTGTA	TTATGTTAATT	C → D
Ab242	Chromosomal *dif*	AGTTCGCATAA	TGTATA	TTATGTTAAAT	-
Ab825	Chromosomal *dif*	ATGACGCATAA	TGTATA	TTATGTTAAAT	-

a The numbering and sequences of the different pXerC/D core regions are those assigned previously to the sites inferred in *A. baumannii* Ab242 plasmids (Cameranesi et al., 2018). All sequences are drawn in the C → D direction, and encompass the 11-bp left half-site recognized by the XerC recombinase, the 11-bp right half-site recognized by the XerD recombinase, and the 6-bp cr spacer separating them. The relative orientations (polarity) between sites found in a same plasmid molecule are indicated in the last column. The designation of hybrid pXerC/D sites product of SSR between pairs indicates the sources of the C- and D- half-sites on each novel site. For comparison purposes the nucleotide differences between sites are highlighted in red to facilitate the visualization of the SSR. The core sequences of the chromosomal *dif* sites of the Ab242 and Ab825 strains, retrieved from WGS data, are shown in the last two lanes.

b See Figure 1.

**Table 2:** Summary of the PCR evidence for the coexistence of different plasmid structural variants containing inversions and fusions resulting from SSR mediated by active pairs of pXerC/D sites in clonal populations of the different *Acinetobacter* strains analyzed.^a^

	*Acinetobacter baumannii* strain or *Acinetobacter* transformant				
XerC/D site	Ab825	Ab242	AnM2/ pAb825^b^	AnM2/ pAb242^c^	Ab17978/pAb242^d^
D2/D2	+	+	+	+	+
C7/D7	w	+	+	+	+
C2/D7	+	+	+	+	+
C7/D2	+	+	+	+	+
C9/D7	+	+	+	+	n.a.
C7/D9	+	+	+	+	n.a.
C9/D9	+	+	+	+	n.a.

+, detected amplification fragment containing the indicated site. n.a., not assayed.

a See Supplementary Figure S4 for PCR details and analysis of the amplification fragments.

b *A. nosocomialis* M2 transformed with Ab825 plasmids.

c *A. nosocomialis* M2 transformed with Ab242 plasmids.

d *A. baumannii* ATCC17978 transformed with Ab242 plasmids.

In summary, the comparative sequence analysis of the plasmids housed by the Ab242 and A825 strains assembled from WGS data analysis indicated that they shared extensive sequence identity as a whole, but also disclosed significant differences between strains including variations in the structural organization of their carbapenem-resistance plasmids and a complete IS*26*-based Tn*aac(3)*-IIa transposon-like structure in pAb825_36 (Figure 1). Moreover, these comparisons pointed to pXerC/D-mediated SSR as one of the main agents behind these structural differences.

### Reversible DNA inversions mediated by recombinationally-active pairs of pXerC/D sites in Ab825 and Ab242 carbapenem-resistance plasmids

The presence of the pXerC/D sites predicted in the carbapenem resistance plasmids harbored by the Ab825 and Ab242 strains (Figure 1) was confirmed by PCR procedures using as templates whole plasmid mixtures extracted from clonal cultures of these strains (see Table 2 for a summary and Supplementary Figure S4 for details). These assays indicated the expected presence of plasmids containing C2/D7 and C7/D2 sites among Ab825 plasmids, and C2/D2 and C7/D7 sites among Ab242 plasmids. Notably, the use of appropriate primer combinations indicated the presence also of plasmid variants containing C2/D2 and C7/D7 sites among Ab825 plasmids, and variants containing C2/D7 and C7/D2 sites in Ab242 plasmid extracts (Table 2). These results indicated that PSVs containing inversions of the DNA region delimited by the recombinationally active pair C2/D2||C7/D7 (or the pair C2/D7||C7/D2) coexisted in the corresponding plasmid extracts. Based on these results, we define here as pAb242_25 In (Xer2/7; Figure 1C) the pAb242_25 structural variant detected in Ab242 clonal cultures that resulted from an Inversion mediated by the recombinationally-active pair composed of the oppositely-oriented sites C2/D2 and C7/D7 (Xer2/7).

**Figure 2:**
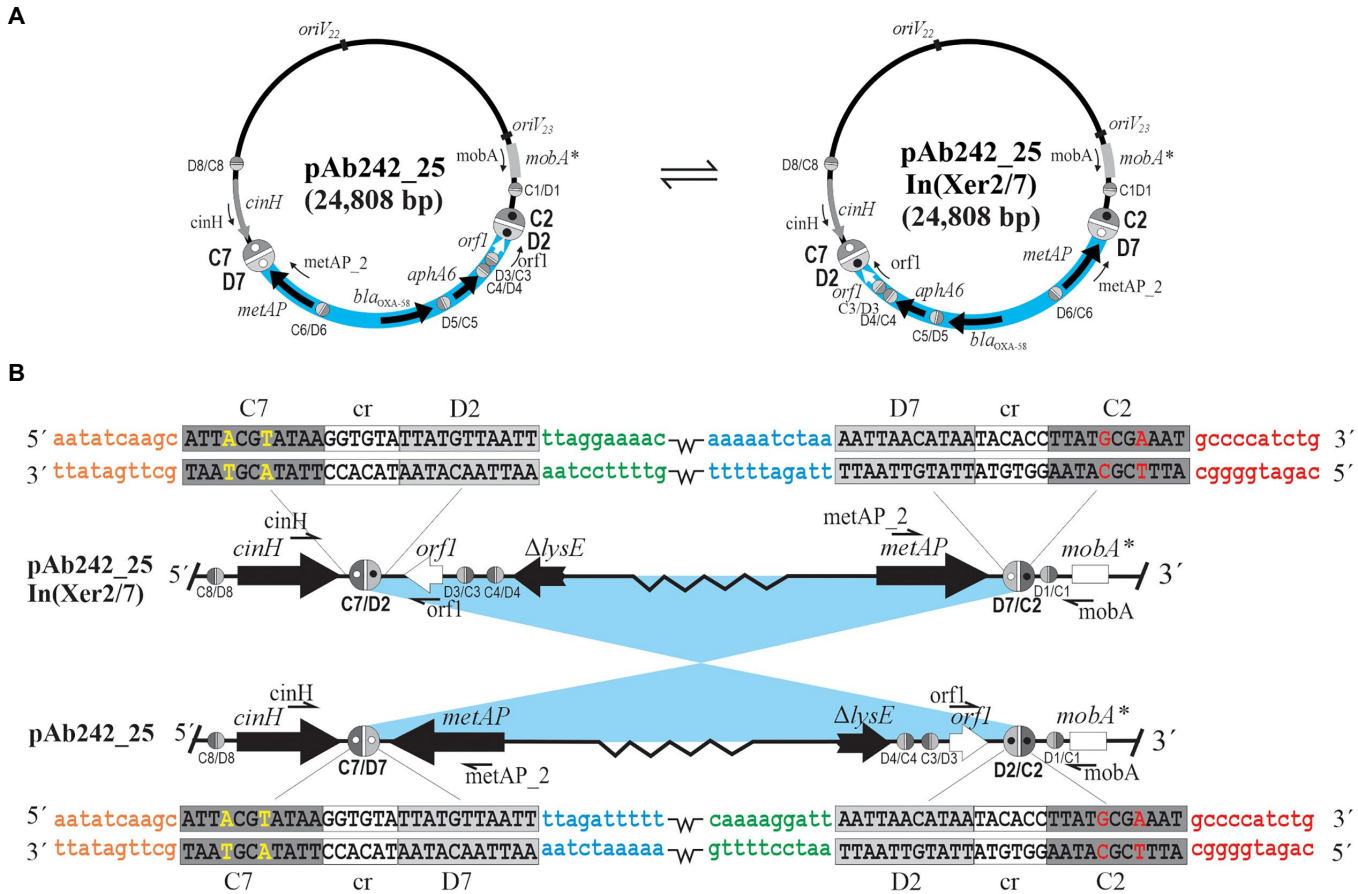
DNA inversions resulting from site-specific recombination between oppositely-oriented sister pairs of pXerC/D sites located at the borders of *bla*_OXA-58_-and *aphA6*-containing adaptive modules in *Acinetobacter* plasmids. **(A)** SSR between the oppositely-oriented sister pair formed by the C2/D2 and C7/D7 sites located in plasmid pAb242_25 mediate the reversible inversion of the intervening sequences between them, thus generating a structural variant designated here as pAb242_25 In(Xer2/7). In turn, the reverse SSR reaction between the resulting oppositely-oriented C2/D7 and C7/D2 “hybrid” sites can regenerate the original pAb242_25 structure. These two structural variants co-exist in clonal populations of the *A. baumannii* strains analyzed, as well as in clonal populations of other *Acinetobacter* strains transformed with plasmids carrying the adaptive module present in these *A. baumannii* strains. The DNA region carrying the *bla_OXA-58_*- and *aphA6*-containing RM is highlighted in pale blue, with the orientation of the genes indicated in each case. The different pXerC/D-like recognition sites are indicated as ovals (not drawn to scale), with the C and D binding regions depicted as dark and light gray semi-ovals, respectively. The active sister pairs mediating inversions have been arbitrarily enlarged, and additional open and closed inner circles within have been incorporated to facilitate visualization of the recombinatorial events. The PCR primers used to identify the presence of specific pXerC/D sites (see Supplementary Table S1 for details) are indicated. The *oriV22* and *oriV23* rectangles denote the location of the iteron regions of the two different replication modules predicted in these plasmids. **(B)** Enlarged vision of the SSR reactions conducing to the reversible inversions described above. The lowercase colored letters show the DNA sequences located at the immediate vicinities of each specific half-site forming the pXerC/D core (10 bp each), and have been included to facilitate visualization of the SSR events. Note that the sequences of the cr and the XerD binding regions are identical for all recombining sites, while the XerC binding regions show some differences between sites (highlighted in uppercase colored letters). The figure is not drawn to scale.

The overall results suggested that a dynamic interconversion of PSVs resulting from reversible SSR events mediated by specific sister pairs of oppositely-oriented pXerC/D sites, in this case C2/D2||C7/D7 and C2/D7||C7/D2 (Figure 2), is in operation in both Ab825 and Ab242 cells.

We next used a transformation approach to verify the existence of the dynamic interconversion of PSVs postulated above. The rationale behind (see Supplementary Figure S2) consisted in the generation of clonal lineages of a model *Acinetobacter* strain (e.g., *A. nosocomialis* M2, AnM2). Each clonal lineage is derived from an individual cell transformed with a single PSV carrying the *bla*_OXA-58_-containing RM, from the different structural variants present in a plasmid mixture extracted from a clonal population of the *A. baumannii* strain under study. The replication of a single incoming plasmid in the new host cell would allow growth in selective medium and the subsequent generation of a clonal population derived from this original event. If a dynamic interconversion of PSVs resulting from reversible SSR events mediated by specific sister pairs of oppositely-oriented pXerC/D sites existed in the cells of the *A. baumannii* strain analyzed, this state could be re-established in a clonal population of the new *Acinetobacter* host and the different co-existing PSVs could therefore be detected by PCR assays.

Competent AnM2 cells were thus transformed with whole plasmids extracted from the Ab825 strain (AnM2/pAb825) or the Ab242 strain (AnM2/pAb242), followed by selection on LB solid medium containing IPM. Individual colonies of AnM2/pAb825 or AnM2/pAbAb242 testing positive for the *bla*_OXA-58_ gene were then used to generate clonal cultures, from which plasmids were extracted and subjected to PCR analysis as indicated above. Using these procedures, we could systematically reproduce in these transformants the PCR results obtained above using plasmids extracted from clonal cultures of Ab825 or Ab242 cells, that is, the co-existence of PSVs containing C2/D2||C7/D7 and C2/D7||C7/D2 sites also in AnM2/pAb825 and in AnM2/pAb242 cells (Table 2; Supplementary Figure S4). Moreover, similar results were obtained for Ab17978 cells transformed with Ab242 plasmid extracts (Ab17978/pAb242). This indicated that a dynamic interconversion of PSVs containing inversions of the DNA regions delimited by the pairs C2/D7||C7/D2 and C2/D2||C7/D7 and resulting from reversible SSR events mediated by these sites could be reproduced in clonal populations of the new hosts (Figure 2).

### Detection of reversible plasmid fusions in Ab825 and Ab242 cells mediated by recombinationally-active pairs of pXerC/D sites

We experimentally verified previously (Cameranesi et al., 2018) the fusion of two Ab242 plasmids, pAb242_25 and pAb242_12, into a co-integrate designated pAb242_37 as the result of a SSR event mediated by the pXerC/D sites C7/D7 and C9/D9 (see Figure 1A for the corresponding structure). Moreover, we found that pAb242_37 could also resolve in the new host by employing a recombinationally active pair now formed by the C7/D9 and C9/D7 hybrid sites formed during the previous fusion.

We designed different PCR primers based on these results (Supplementary Table S1) aimed to test the presence of similar co-integrates (and their resolution products) in the plasmid extracts of AnM2/pAb242 and AnM2/pAb825 cells employed above. Hybrid C9/D7 and C7/D9 sites were both detected in AnM2/pAb242 plasmid extracts (Table 2; Supplementary Figure 4F, first two lines at the left), indicating the presence of the pAb242_37 co-integrate in these cells. Moreover, PSVs containing the C9/D9 site (Supplementary Figure 4F, third line at the left) and the C7/D7 site (Supplementary Figure 4F, second lane) were also detected in the same plasmid extracts. The coexistence of both the co-integrate and its resolution products in these cells reinforced the notion (Supplementary Figure S2) that the pAb242_37 co-integrate represented the incoming plasmid form with the ability to replicate and establish in a new AnM2 host cell, and that this co-integrate could also undergo resolution in these cells *via* an intramolecular SSR event mediated now by the directly-oriented hybrid pair C9/D7||C7/D9 (Supplementary Figure S5). Similar observations were made using AnM2/pAb825 plasmid extracts, indicating the co-existence in these extracts of PSVs that included a co-integrate variant containing the C9/D7 and C7/D9 hybrid sites as well as individual plasmids carrying the C9/D9 and C7/D7 sites product of the resolution of this co-integrate (Table 2; Supplementary Figures S4F, S4C, respectively). Again, these observations support the existence of a dynamic state of resolution and re-synthesis of similar co-integrates in both Ab825 and Ab242 cells (Supplementary Figure S5).

Contrary to the above results, PCR assays aimed to detect the presence of the C14/D14 site and neighboring regions using Ab825 plasmid extracts as templates (Supplementary Table S1) consistently failed, in sharp contrast with the amplification bands obtained for the C4/D14 and C14/D4 sites using the same extracts and different combinations of the same primers (Supplementary Figures S3, S4G). A successful detection of the C14/D14 site in Ab825 plasmid mixtures would have implicated the presence of a pAb242_9 circular plasmid structure, and therefore that pAb825_36 (Figure 1D) could be resolved in these cells into its putative plasmid constituents in a SSR event mediated by the directly-oriented C4/D14 and C14/D4 sites. It is worth noting that these sites contain identical XerC- and identical XerD- half-sites, but differed in 2 out of 6 positions in the cr spacer sequence (Table 1). This resolution, if taking place, probably occurs at a very low rate as compared to the other SSR events described above which could be reproducibly detected by the PCR assays employed.

The overall observations thus support the existence of a dynamic interconversion state of PSVs involving reversible intramolecular inversions and fusions/resolutions mediated by recombinationally active pairs of pXerC/D sites sharing identical GGTGTA cr spacers in both Ab825 and Ab242 cells. Moreover, this dynamic interconversion of PSVs could be reinstalled in the cells of other *A. baumannii* strains or other *Acinetobacter* species after transformation with a PSV carrying the *bla*_OXA-58_-containing RM and possessing the capability to establish in the new host.

### Structural comparisons between *A. baumannii* Ab242 and Ab825 plasmids with similar plasmids described in other Acinetobacter strains provide clues on the evolution of novel Rep_3 replicons endowed with blaOXA58-containing resistance modules

**Figure 3:**
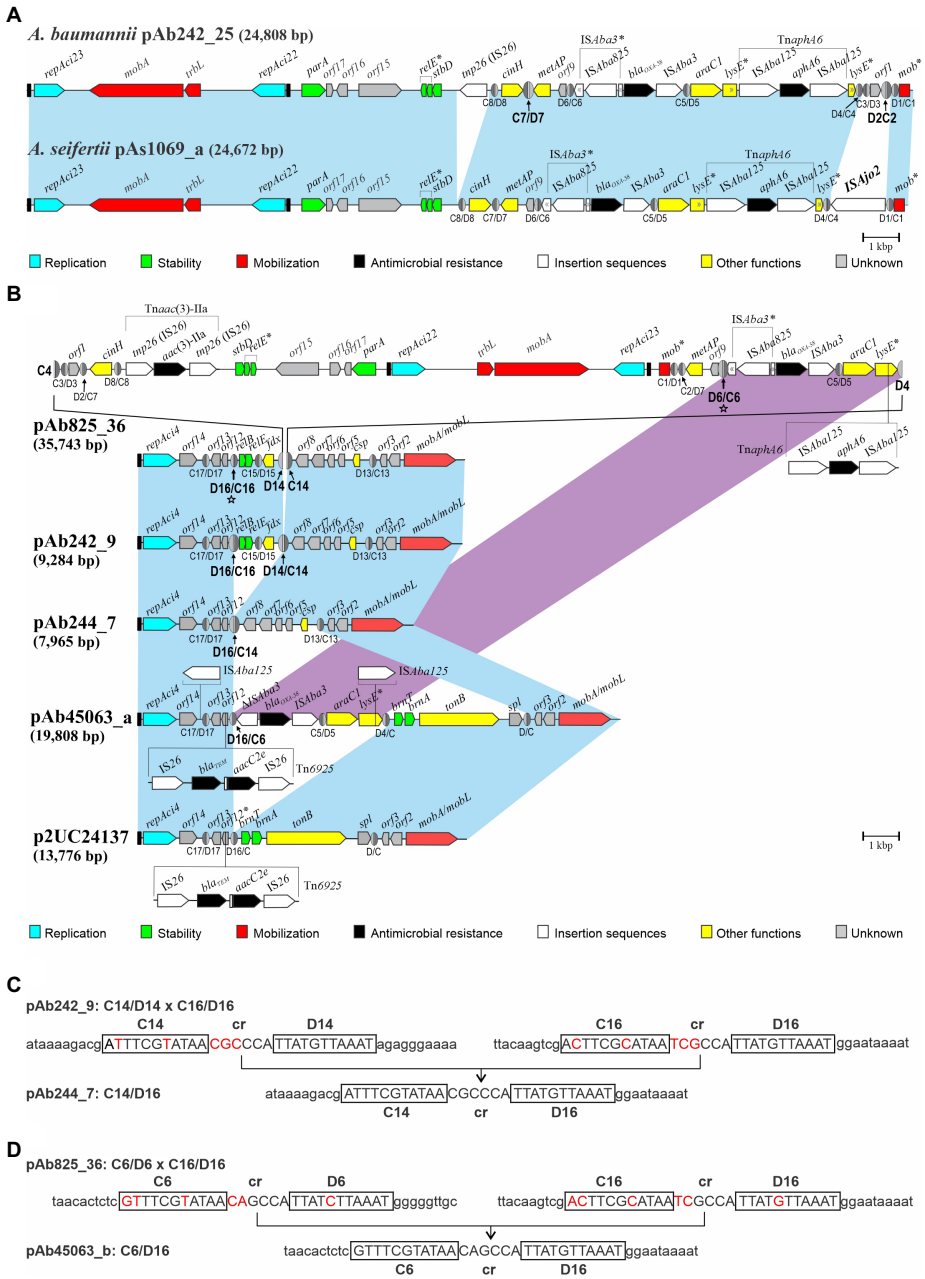
Structural comparisons between *bla*_OXA-58_-containing Rep_3 plasmids carried by *Acinetobacter baumannii* Ab242/Ab825 strains with sequence-related plasmids deposited in databases described in other *Acinetobacter* strains. **(A)** Comparisons of *bla_OXA-58_*-containing pAb242_25 with a similar *repAci23*- and *repAci22*-containing bi-replicon carried by the *Acinetobacter seifertii* 1,069 strain isolated in Brazil, pAs1069_a (GenBank accession number MK323040). The plasmids share 95% total sequence identity, all containing almost identical RMs encompassing *bla_OXA-58_*- and *aphA6*-resistant genes which show the same orientation in pAs1069_a as compared to pAb242_25. However, pAs1069_a contains an extra IS*Ajo2*-like element at the Tn*aphA6*-proximal end of the RM (1,482 bp; nucleotide positions 22,311 to 23,792), an insertion that is concomitant with the deletion of around 490 bp containing the C2/D2 and C3/D3 sites in pAb242_25. For other details on plasmid structures see the legend to Figure 1. **(B)** Comparisons of the pAb825_36 tri-replicon structure characterized in this work with different plasmids containing backbones endowed with a *repAci4* replication module and *mobA*/*mobL* genes described in other *A. baumannii* CC15 strains. The light-blue shaded background interconnecting the different plasmid structures denote shared regions of sequence homology between plasmids. ISs or IS-based transposons found in a given plasmid but not in others within these otherwise homologous regions were represented either above or below the corresponding structures. The magenta-shaded background denotes homologous regions carrying similar pXerC/D-bounded *bla_OXA-58_*-containing RMs between pAb825_36 and pAb45063_a. The pXerC/D sites constituting sister pairs mediating SSR events, or resulting from these events, have been arbitrarily enlarged. The directly-oriented C6/D6 and C16/D16 sites in pAb825_36 (labeled with stars ★), proposed here to mediate an intramolecular SSR event conducing to a pAb45063_a-like plasmid as one of the resolution products, have been also arbitrarily enlarged. GenBank accession numbers: pAb244_7: MG520098; pAb45063_a: MK323042. For other details on plasmid structures see the legend to Figure 1. **(C)** Sequence comparison evidence indicating the existence of an intramolecular SSR resolution event in pAb242_9 mediated by the directly-oriented C14/D14 and C16/D16 sites conducing to the generation of pAb244_7 containing a C14/D16 (hybrid) site. The pXerC/D core sequences of each site are displayed in uppercase letters, where the XerC and XerD half-sites have been additionally boxed. For a better visualization of the SSR event the nucleotide differences between core sites on the substrate plasmid have been highlighted in red, and additional sequences (10 bp in each case) at the immediate vicinities of each specific half-site have been included. **(D)** Same, indicating an intramolecular SSR resolution event in a pAb825_36-like substrate plasmid mediated by the directly-oriented C6/D6 and C16/D16 sites resulting in the generation of a pAb45063_a-like plasmid containing a C6/D16 (hybrid) site.

A BlastN search against the nucleotide GenBank database (as of August 15, 2022) using as query the Ab242_25 sequence (KY984047) identified a similar plasmid, pAs1069_a (accession number MK323040) housed by a carbapenem-resistant *Acinetobacter seifertii* clinical strain isolated in Brazil (Matos et al., 2019). pAb242_25 and pAs1069_a share 95% total sequence identity, including the presence and orientation of the *bla*_OXA-58_- and Tn*aphA6*-containing RM in the corresponding molecules (Figure 3A). However, one remarkable difference between these two plasmids is the number of pXerC/D sites found at the Tn*aphA6*-proximal end of the corresponding RMs, which were reduced to two sites in pAs1069_a as compared to the four sites located in the equivalent region of pAb242_25. It is worth noting that pAs1069_a contains in this region an IS*Ajo2*-like element, and sequence comparison analysis indicated that the acquisition of this element was concomitant with the deletion of a fragment of around 490 bp that in pAb242_25 encompasses the C2/D2 and C3/D3 sites (Figure 3A). Among the seven pXerC/D sites located at the borders of the *bla*_OXA-58_- and Tn*aphA6*-containing RM in pAb242_25, only the C7/D7 and C2/D2 sites share identical GGTGTA cr sequences (Table 1, see also Cameranesi et al., 2018). The selection of the insertion of this IS*Ajo2* element at this region in pAs1069_a, by removing the C2/D2 site, is most probably associated then with an impairment of the reversible inversion ability of the DNA fragment encompassing the carbapenem RM.

The use of pAb242_12 (KY984046) as a query retrieved no *repAci21*-containing plasmids carrying *bla*_OXA-58_ genes, with the exception of the pAb242_37 multi-replicon in which a pXerC/D-bounded pAb242_12 domain is inserted (Figure 1). On the contrary, the use of pAb242_9 (KY984045) identified, besides the pXerC/D-bounded pAb242_9 domain carried by the pAb825_36 tri-replicon (Figure 1), a series of plasmids sharing backbones also composed of a *repAci4*-based replicon and a *mobA*/*mobL* module (Figures 3B,C). It is worth noting that these plasmids were carried by *A. baumannii* CC15 strains isolated in Argentina, Brazil, and Chile (Matos et al., 2019; Cameranesi et al., 2020; Brito et al., 2022). Among them pAb244_7, the smallest plasmid of this group, differs from pAb242_9 in an approximately 1.3 kbp DNA region carrying genes for a *relBE* system and ferredoxin (*fd*; Figure 3B). This region is bordered by two directly-oriented pXerC/D sites, C14/D14 and C16/D16, which differ in 2 out of 11 positions in their XerC-binding regions and in 3 out of 6 positions in their cr spacers (Figure 3C). Yet, the identification of a hybrid C14/D16 site in pAb244_7 strongly suggests that a XerC/D-mediated resolution event occurring in pAb242_9 could be responsible for the loss of this pXerC/D-bounded module.

pAb45063_a, another member of this group, present in an *A. baumannii* CC15 strain isolated in Brazil (Matos et al., 2019), carries an IS*26*-based composite-like transposon endowed with *bla*_TEM-1_ and *aacC*(3)-IIa resistance genes and, most relevantly for this work, a pXerC/D-bounded *bla*_OXA58_-containing RM (Figure 3B). Differences in mobile elements content between the RMs found in pAb45063_a and Ab242/Ab825 plasmids (Figure 1), such as the IS*Aba825* element found upstream of the *bla*_OXA-58_ gene and the Tn*aphA6* transposon inserted within the *lysE* gene in the latter plasmids as compared to the single IS*Aba125* copy interrupting *lysE* in pAb45063_a (Figure 3B), are commonly observed among *Acinetobacter* Rep_3 plasmids carrying similar adaptive modules, and most probably result from the collection of different ISs and transposons during plasmid transit through different bacterial hosts (Cameranesi et al., 2018). Most importantly however, these comparisons provide clues on how the generation of complex multi-replicon co-integrates such as pAb825_36 could also supply the substrates for subsequent XerC/D-mediated structural rearrangements that eventually evolve in novel replicon structures carrying *bla*_OXA-58_-containing RMs. In principle, a SSR resolution event involving the directly-oriented C6/D6 and C16/D16 sites located in pAb825_36 as a sister pair (Figure 3D) could have generated two separate plasmids, one *repAci23*- and *repAci22*-containing bi-replicon and another replicon containing a *repAci4*- and *mobA*/*mobL*-backbone now carrying a *bla*_OXA-58_-containing RM similar in structure to pAb45063_a (Figure 3B).

## Discussion

We sought to analyze in this work whether and how pXerC/D-mediated recombination contributed to the generation of structural diversity between carbapenem resistance plasmids harbored by the *A. baumannii* strains Ab242 and Ab825, two phylogenetically- and epidemiologically-closely related CC15 strains isolated from hospitalized patients in the same public healthcare center in Argentina. These two strains harbored similar Rep_3 plasmids carrying almost identical RM endowed with a *bla*_OXA-58_ gene accompanied with a Tn*aphA6* composite transposon, a structure associated to seven non-identical pXerC/D sites at their borders. Comparative sequence analysis of Ab242 and Ab825 plasmids assembled on the basis of WGS data (Figure 1) indicated significant structural variations between them, and pointed to XerC/D-mediated SSR as the main agent behind these differences.

We developed a series of methodologies to experimentally verify that SSR events mediated by pXerC/D sites represented the cause of the structural differences observed between Ab242 and Ab825 plasmids carrying the above-described RM. These procedures allowed us to disclose the existence of *bona fide* pairs of recombinationally-active pXerC/D sites in these plasmids, some mediating reversible intramolecular inversions and others reversible plasmid fusions/resolutions. Among them, the pair formed by the oppositely-oriented C2/D2 and C7/D7 sites, and its SSR products the (hybrid) C2/D7 and C7/D2 pair, mediated reversible intramolecular inversions of the RM in both strains. In turn, the pair formed by the C7/D7 and C9/D9 sites mediated intermolecular fusions, and that formed by its SSR products the (hybrid) C7/D9 and C9/D7 sites mediated intramolecular resolutions. These events strongly suggested that a dynamic interconversion of sequence-related PSVs is in operation in clonal populations of the Ab242 and Ab825 strains analyzed. Moreover, this reversible shuffling of plasmid structures could be reproduced in cells of model *Acinetobacter* strains including *A. nosocomialis* M2 and *A. baumannii* ATCC17978 in laboratory transformation assays. All of the *A. baumannii* pXerC/D pairs identified here as mediating reversible SSR events contained identical GGTGTA cr sequences separating the XerC from the XerD half-sites (Table 1), sharing characteristics with pairs classified as relaxed or unconstrained in other models (Cornet et al., 1994).

The findings that *Acinetobacter* plasmids contain pXerC/D sites capable of conforming recombinationally active pairs mediating reversible remodeling of their structures significantly impact the possibilities of rapid adaptation of their eventual hosts to varying environments, the evolution of plasmid structures, and the dissemination of *bla*_OXA-58_ genes among both *Acinetobacter* and non-*Acinetobacter* populations. In principle, this dynamic shuffling of plasmid structures could provide an eventual *Acinetobacter* host fitness advantage to confront the different selective pressures that the population may encounter during growth. In this context, a number of other bacterial species have been found capable of generating genetically- and phenotypically- heterogeneous subpopulations by employing reversible SSR mechanisms (Didelot et al., 2016; Andam, 2019; García-Pastor et al., 2019; Jiang et al., 2019; Trzilova and Tamayo, 2021). The degree of diversity in these cases solely depends on the number of phase-variable loci, and this process has a profound impact on the survival or fitness capacity of the population as a whole. A valuable asset of reversibility is the ability to rapidly restore genotypic and phenotypic heterogeneity after a bottleneck, a common situation for a bacterial pathogen such as *A. baumannii* that frequently transits from the human host to the environment and vice versa, especially in the highly selective conditions of the clinical setting.

The dynamic interconversion of plasmid structural variants could be also viewed as a trial- and-error game for the long-term adaptation of an eventual *Acinetobacter* host to a specific niche or to long-term exposures to particularly challenging conditions. The majority of bacterial genes are encoded on the leading strand of DNA replication such that transcription is co-directional with fork movement, preventing the deleterious consequences of head-on replication-transcription conflicts (Schroeder et al., 2020). Inversions that result in head-on conflicts promote the selection of mutations, including insertions/deletions and base substitutions depending on genetic context, leading to proposals that the generation of such conflicts potentiates bacterial evolvability (Merrikh and Merrikh, 2018). The dynamic state of DNA inversions and fusions/resolutions reported here in *Acinetobacter* plasmids, by promoting temporary head-on conflicts, could then accelerate the mutation rate in certain plasmid regions with lasting consequences for the evolution of these mobile elements. For instance, and depending on host and environmental pressures, mutational events could be selected that “freeze” a given structural variant thus allowing, for instance, continuous expression of a gene whose product is in extended demand. In this context, the several pXerC/D sites located at the borders of the plasmid-borne *bla*_OXA58_-containing RM studied here (Figure 1) provide different alternatives for selection, which may range from complete deletions that abolish the possibilities of recombination (and thus shuffling) to subtle mutations that impede (or increase) the activity of a given pXerC/D pair. The comparisons shown in Figure 3A indicating the IS*Ajo*2-mediated elimination in *A. seifertii* plasmid pAs1069_a of a site composing a recombinationally active pXerC/D pair in Ab242/Ab825 plasmids (Figure 1), provide an example of the above. These observations additionally suggest that the position (or orientation) of the *bla*_OXA-58_-containing RM in the *Acinetobacter* plasmid molecule is subjected to selection pressures, and that the selection of a particular orientation most probably depend on the fitness provided to a particular plasmid or plasmid/host partnership in a given environment.

The reversible SSR events mediated by pXerC/D sites reported here may have played important roles in facilitating the wide dissemination of *bla*_OXA-58_ genes among plasmids carried by *Acinetobacter* species, and also among other bacterial populations (Zarrilli et al., 2008; Carattoli, 2013; Evans and Amyes, 2014; Fu et al., 2014; Zander et al., 2014; Da Silva and Domingues, 2016; Hamidian and Nigro, 2019; Bonnin et al., 2020; Wang et al., 2020; Alattraqchi et al., 2021; Hamidian et al., 2021; Liu et al., 2021; Brito et al., 2022; Jones et al., 2022). It is noteworthy in this context the generation of multi-replicons resulting from pXerC/D-mediated SSR events in both the Ab242 and Ab825 strains (Figure 1). Multi-replicon plasmids have been described in many *Acinetobacter* members by different authors, suggesting selective advantage(s) for these ensembles (Carattoli, 2013; Shintani et al., 2015; Cameranesi et al., 2018, 2020; Hamidian and Hall, 2018; Dionisio et al., 2019; Mindlin et al., 2020; Salgado-Camargo et al., 2020; Alattraqchi et al., 2021; Jones et al., 2022). A reversible fusion of different replicons, by expanding the host range or by providing stability (or other functions) to the ensemble, could play important roles in the establishment of the different plasmid components in a given host (Dionisio et al., 2019). In this context, we observed that a co-integrate between pAb242_25 (or a pAb242_25-related plasmid) and pAb242_12 was the form that could successfully establish in different *Acinetobacter* hosts including *A. nosocomialis* M2 and *A. baumannii* ATCC17978 in laboratory transformation assays (Table 2). The presence of pXerC/D sites in *bla*_OXA-58_-containing resident plasmids also opens the possibility of reversible co-integrate formation with newly incoming conjugative plasmid(s) endowed with pXerC/D sites, thus allowing the transfer of the cargo plasmid by conduction. Moreover, a rapid resolution of the co-integrate in the new host by the reverse SSR reaction may facilitate the rapid segregational loss of the conjugative, energy-demanding counterpart in the progeny especially under antimicrobial selection pressure for the cargo plasmid (Dionisio et al., 2019).

The generation of complex multi-replicon structures endowed with *bla*_OXA-58_-containining RMs, by generating new potential pairs of recombinationally active pXerC/D sites capable of mediating further intramolecular resolution events, can also facilitate the evolution of novel replicons carrying *bla*_OXA-58_-containing RMs (Figure 3B). This mechanism, added to structural rearrangements generated by subsequent pXerC/D-mediated SSR events and the insertions of different ISs and transposons, could explain the evolution of novel carbapenem-resistance plasmids in the local *A. baumannii* CC15 population.

The different pXerC/D sites inferred in Ab242/Ab825 plasmids show some sequence differences between them at the level of the XerC- and also the XerD-half sites on the core, as well as in the cr spacer separating these regions (Cameranesi et al., 2018). The importance of homology between the core regions of the two recombining sites has not been clearly established for the *E. coli* XerC/D system (Rajeev et al., 2009), and little information exists on the *A. baumannii* XerC/D system. However, recent studies (Lin et al., 2020) indicated similar functions for this SSR system as compared to its *E. coli* counterpart. Our results (Table 2) indicated that reversible recombination could occur between pairs of sites sharing identical GGTGTA cr spacer sequences, even when these recombining sites display some nucleotide differences at the level of the XerC- and also XerD- half-sites. On the contrary, reversible recombination could not be detected for the C4/D14 and C14/D4 sites found in pAb825_36, which contained identical XerC- and identical XerD- half-sites but differed in 2 out of 6 positions in the cr spacer sequence (Table 2). This suggests that the reversibility potential of SSR reactions mediated by pXerC/D sites depends on the cr sequences of the pair involved. In this context, XerC/D-mediated recombination between plasmids containing *dif* and *psi* core sites sharing almost identical XerC- and also XerD- half-sites but differing in their cr sequences in 4 out of 6 positions occurred in *E. coli* cells at very low rates, as judged by the low frequency of colonies carrying plasmids with *dif*/*psi* hybrid sites obtained (Cornet et al., 1994). It was proposed that the XerC/D recombinases could mediate in these cases first strand exchanges but not the subsequent resolutions of the Holliday junction intermediate (HJ) formed, which would then be processed by XerC/D-independent cellular mechanisms (Cornet et al., 1994; Rajeev et al., 2009; Ramirez et al., 2014; Lin et al., 2020). Similarly, it remains possible that in the absence of complete cr identity between sites the *A. baumannii* XerC/XerD recombinases could mediate only first strand exchanges followed by HJ resolution by other mechanisms, making the reaction essentially irreversible. The selection of these infrequent events probably depends on the fitness provided by the resulting plasmid ensemble.

How far in time could we trace the existence of pXerC/D sites in *A. baumannii*? Although environmental *A. baumannii* strains of non-clinical origin are extremely rare (Antunes et al., 2014), we could recently reclassify as *A. baumannii* (Repizo et al., 2017, 2020) a collection strain, *Acinetobacter* sp. NCIMB8209, isolated before 1944 from the enriched microbiota responsible for the aerobic decomposition of the desert shrub guayule in a process aimed for the industrial production of natural latex (Allen et al., 1944). Expectedly from an environmental isolate obtained around or before 1944, WGS analysis of the NCIMB8209 genome indicated a general absence of antimicrobial resistance genes. Most relevantly for this work, however, this analysis also indicated the presence of a large Rep_3 plasmid of 133,709 bp designated pAbNCIMB8209_134 (GenBank accession CP028139.1) in which we detected a pair of directly-oriented pXerC/D sites, namely ATTTCGTATAAGGTGTATTATGTTAATT (positions 48,301 to 48,328) and ATTTCATATAAGGTGTATTATGTTAATT (positions 49,996 to 50,016), flanking a gene cluster composed of a toxin-antitoxin gene cluster (C4X49_18715/C4X49_18720) accompanied by an exonuclease subunit gene (C4X49_18710). Notably, these two sites also shared identical GGTGTA cr sequences. Multiple pXerC/D sites have also been identified in the pAB1 and pAB2 Rep_3 plasmids (Balalovski and Grainge, 2020) present in the *A. baumannii* ATCC17978 collection strain isolated from a clinical source around 1950 (Piechaud and Second, 1951). These inferences indicated that Rep_3 plasmids carrying pXerC/D-bounded genes were already present in the *A. baumannii* population before the massive anthropogenic introduction of antimicrobials by the middle of last century (Antunes et al., 2014; Baquero et al., 2021). GGTGTA cr sequences can also be found among 6 out of the 17 non-identical pXerC/D sites identified by us among Ab242/Ab825 plasmids (Cameranesi et al., 2018), and between many of the multiple pXerC/D sites described in other plasmids of many *Acinetobacter* (and even non-*Acinetobacter*) strains isolated in different periods and from different sources, both clinical and environmental (Fu et al., 2014; Blackwell and Hall, 2017; Brovedan et al., 2019; Matos et al., 2019; Mindlin et al., 2019; Balalovski and Grainge, 2020; Bonnin et al., 2020; Salgado-Camargo et al., 2020; Wang et al., 2020; Alattraqchi et al., 2021; Hamidian et al., 2021; Liu et al., 2021; Jones et al., 2022). Although it remains to be established what other factors influence the feasibility (or reversibility) of SSR events between sites sharing identical cr sequences, it is likely that some of these sites participate in the remodeling and evolution of *Acinetobacter* plasmids in the eventuality that they collide in the same cell, with the concomitant accelerated dissemination of the resistance determinants they bound. The whole process could probably be viewed then more adequately from the units-of-evolution perspective (Baquero et al., 2021).

Further work is in progress to address many of the interrogates posed above, including the influences of the different *Acinetobacter* pXerC/D core sequences and genetic context on the feasibility and directionality of the recombination reaction, as well as the detailed roles of the XerC and XerD recombinases in the recombination process.

## Author's Note

Part of the results of this work have been previously presented in the following International Meetings:

1. A. M. Viale, M. Cameranesi, A. Limansky, G. Repizo, and J. Morán-Barrio. Site-specific recombination at XerC/D sites as mediators of plasticity among *Acinetobacter baumannii* plasmids carrying carbapenem- and aminoglycoside-resistance genetic modules. Plasmid Biology Symposium, Seattle, USA, August 2018.
2. M. M. Cameranesi, J. Morán-Barrio, A. S. Limansky, G. D. Repizo, A. M. Viale. Detection of active pairs of XerC/D recognition sites mediating fusions and inversions in *Acinetobacter baumannii* plasmids carrying OXA-58 carbapenemase adaptive modules. 12th International Symposium on the Biology of Acinetobacter, Frankfurt, Germany, September 2019.

## Data availability statement

The datasets presented in this study can be found in online repositories. The names of the repository/repositories and accession number(s) are as follows. GenBank accession numbers for the Ab242 and Ab825 plasmids included in this work are: pAb242_25, KY984047; pAb242_12, KY984046; pAb242_9, KY984045; pAb825_36, MG100202; pAb25_12, MG100203. GenBank accession number for plasmid pAb244_7 is MG520098. GenBank accession numbers for the sequences of different PCR amplification fragments containing the particular pXerC/D sites and corresponding genetic contexts presented in this study (Supplementary Table S1) are as follows: C2/D2 site, ON060992; C2/D7 site, ON060990; C7/D2 site, ON060991; C9/D7 site, OM876186; C7/D7 site, ON060993; C7/D9 site, OM876185; C9/D9 site, ON060994.

## Author contributions

AV, MC, JM-B, and AL conceived and designed the work. MC, LG, and RS performed the experimental work. MC, AV, and JM-B conducted the bioinformatic analysis. AV, MC, LG, RS, JM-B, and AL analyzed and discussed the data. AV wrote the manuscript. All authors contributed to the article and approved the submitted version.

## Funding

This work was supported by grants from CONICET PIP 11220170100377CO to JM-B and AV, Agencia Nacional de Promoción Científica y Tecnológica (ANPCyT) PICT-2017-3536 to JM-B, PICT-2019-00074 to AL, and Ministerio de Ciencia, Tecnología e Innovación Productiva, Provincia de Santa Fe, Argentina, to AV and AL. MC is a former fellow of CONICET, LG is a fellow of ANPCyT, RC is a fellow of CONICET, JM-B, and AV are Career Researchers of CONICET, and AL is a Researcher of the National University of Rosario, Argentina.

## Conflict of interest

The authors declare that the research was conducted in the absence of any commercial or financial relationships that could be construed as a potential conflict of interest.

## Publisher’s note

All claims expressed in this article are solely those of the authors and do not necessarily represent those of their affiliated organizations, or those of the publisher, the editors and the reviewers. Any product that may be evaluated in this article, or claim that may be made by its manufacturer, is not guaranteed or endorsed by the publisher.
