# Supplementary material for: Dynamic state of plasmid genomic architectures resulting from XerC/D-mediated site-specific recombination in Acinetobacter baumannii Rep_3 superfamily resistance plasmids carrying blaOXA-58- and TnaphA6-resistance modules
Source: Front Microbiol. 2023 Feb 9;14:1057608. doi: 10.3389/fmicb.2023.1057608 (PMC9947245; doi:10.3389/fmicb.2023.1057608)
Supplement: Supplementary file 1 [file Data_Sheet_1.PDF]

Supplementary Material

Supplementary Figure S1.

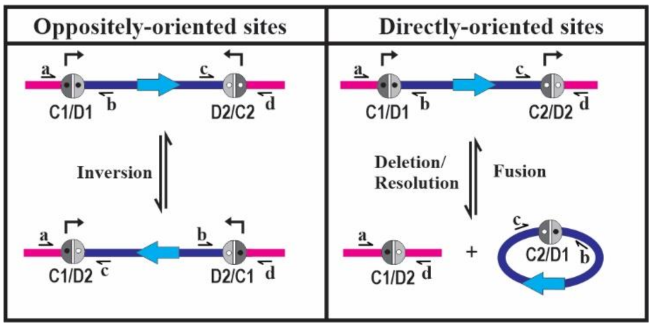

Legend to Figure S1. Genomic structural changes resulting from site-specific recombination mediated by active sister pairs of XerC/XerD sites.

The core XerC/XerD (C/D) recognition site is a 28 bp imperfect palindrome composed of an 11-bp C binding domain (C half-site) recognized by the XerC recombinase, a 6-bp central region (cr or spacer) separating the C and D binding domains and in which strand cleavage and exchange occurs, and an 11-bp D binding domain (D half-site) recognized by the XerD recombinase (Rajeev et al., 2009; Crozat et al., 2014; Balalovski and Grainge, 2020). Left, site-specific recombination (SSR) between a sister pair formed by two oppositely-oriented C/D sites mediates the inversion (denoted by the orientation of the arrows) of the intervening sequences between them. Right, SSR between a sister pair formed by two directly-oriented C/D sites mediates the resolution of the region between sites, which ends up as a circular molecule. In turn, the inverse reaction mediates the integration of a circular DNA molecule into another. If both molecules are circular, a co-integrate structure is formed. An individual pXerC/D site is defined in this work not only by its 28 bp core sequences but also by its location in the plasmid structure, implying the genomic sequences located at the immediate vicinities of its C and D half-sites in the plasmid structure. These features were used in this work not only to confirm the presence of particular pXerC/D sites, but also to identify new (hybrid) sites resulting from recombinatorial exchanges between sister pairs. PCR reactions using combinations of primers hybridizing to specific sequences located at the immediate neighboring regions of each half-site (denoted from a to d in the figure), followed by cloning of the obtained amplicons into the pGem-T Easy vector and further sequencing and comparative analysis, were used for this purpose. pXerC/D sites are shown as ovals with the C and D binding regions depicted as dark and light gray semi-ovals, respectively, with the C-to-D polarity of each site indicated by the arrows above. The small circles inside the C and D motifs and the different colors on the immediate neighboring regions were incorporated to facilitate the visualization of the SSR exchanges.

Supplementary Figure S2.

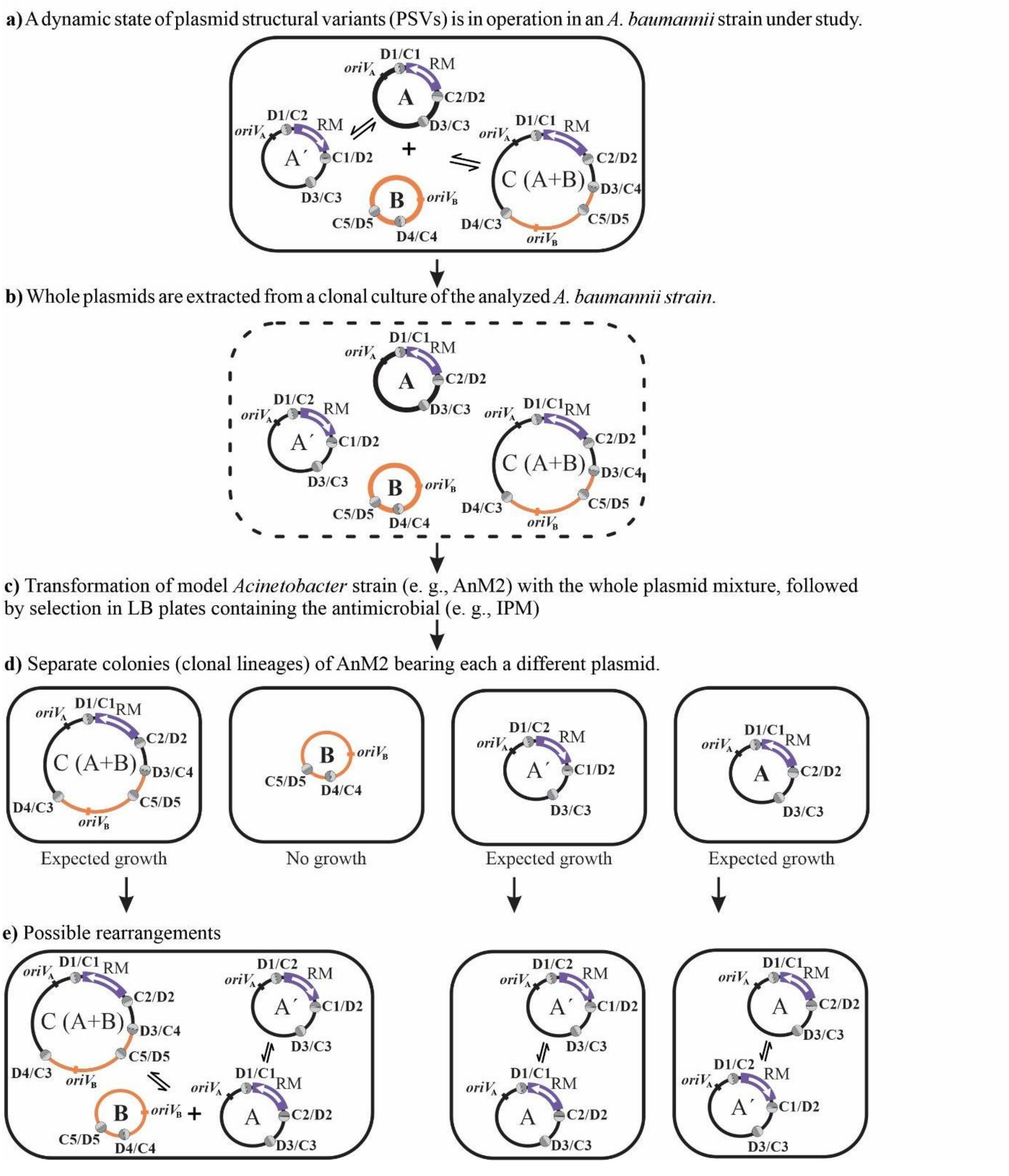

Legend to Figure S2. Schematic representation of the procedures used in this work to detect the presence of structural variants resulting from pXerC/D-mediated site-specific recombination in *Acinetobacter* plasmids.

a) Hypothetical example where A and B represent the predominant plasmid structures assembled on the basis of WGS data analysis and gap closing procedures in a studied *A. baumannii* strain. The two plasmids are arbitrarily shown here as single replicons with the closed rectangles indicating the location of the iteron sequences on the corresponding replication modules. In the example, only plasmid A carries an antimicrobial resistance module (RM), and also multiple non-identical pXerC/D sites (three in A, and two in B, the XerC- (C) and XerD- (D) binding

regions are depicted as indicated in the legend to Fig. S1. Note the opposite orientations between the C1/D1 and C2/D2 sites in plasmid A. If some of the predicted pXerC/D-like sites are capable of conforming active sister pairs mediating reversible SSR events, the plasmid pool of the cell will also include a minor proportion of structural variants of these predominant forms (PSVs) carrying inversions, fusions and/or deletions. Some possible PSVs are exemplified here by C and A´, in which C is a co-integrate of A and B resulting from a (reversible) intermolecular SSR mediated by the sister pair formed by C3/D3 and C4/D4, and A´ is a structural variant of plasmid A in which the sister pair formed by the oppositely-oriented C1/D1 and C2/D2 sites mediated a (reversible) intramolecular inversion of the intervening sequences.
A transformation-based procedure designed to disclose the co-existence of a dynamic state of interconversion of different plasmid structural variants (PSVs) derived from pXerC/D-mediated SSR in clonal *Acinetobacter* cultures is described below.
b-e) Isolation of clonal lineages of *Acinetobacter* model strains transformed with *A. baumannii* carbapenem resistance plasmids. Individual clonal lineages on a model *Acinetobacter* strain such as AnM2 are first obtained after transformation with plasmids extracted from a clonal culture of the *A. baumannii* strain under study carrying carbapenem-resistance plasmids. The transforming plasmid must possess the capability to replicate in the new host and carry a cbarbapenem resistance gene or module (RM), necessary for selection purposes. The transformed cells are incubated in LB agar medium in the presence of the selection agent (e. g., IPM 2 μg/ml), and individual colonies are isolated, characterized, and stored for further analysis. Each separate lineage is derived from an individual AnM2 cell originally transformed with a particular plasmid structural variant (PSV) carrying the RM from the different PSV present in the plasmid mixture. In the example, AnM2 cells individually transformed with co-integrate C, or with plasmid A, or with plasmid A´ (all of which carry the RM) would be expected to generate each a separate clonal lineage in this selective medium. On the contrary, AnM2 cells transformed with plasmid B alone, in which no RM is located, are selected against at this step. Now, if the single incoming resistance plasmid can also reinstate in the new AnM2 host a dynamic interconversion of structural variants involving inversions and/or resolutions/fusions mediated by recombinationally active pairs of pXerC/D sites, the coexistence of these PSVs will be expected in each of the ensuing clonal populations after growing in selective medium. For instance, in the case of an AnM2 cell transformed with A, the co-existence of A and A´ will occur in the ensuing clonal population, and a similar result will be obtained with AnM2 cells transformed with A'. Now, if the transforming plasmid was C, this co-integrate could resolve in its new host into its constituent plasmids A and B, therefore regenerating a dynamic state of resolution and re-fusion in the ensuing clonal population. Moreover, if the reversible inversion mediated by the oppositely-oriented pair of pXerC/D sites interconnecting A with A´ also occurs in this particular lineage, the co-existence of A with A´ will be additionally found in this population. The coexistence of these PSVs can be tested using as template plasmids extracted from this clonal culture and appropriate combinations of PCR primers aimed to independently detect the C co-integrate (sites C3/D4 and C4/D3) and the closed forms of A and B carrying the C3/D3 site and C4/D4 site, respectively. In turn, the co-existence of A with A´ in the population can also be investigated using PCR primers aimed to independently detect the C1/D1, C2/D2, C1/D2 and C2/D1 sites. The verification of the co-existence of various PSVs in clonal cultures of the AnM2 transformants (d) supports in turn the existence of a similar dynamic state of PSVs in the original *A. baumannii* strains from which the plasmids were extracted (a).

Supplementary Figure S3.

A

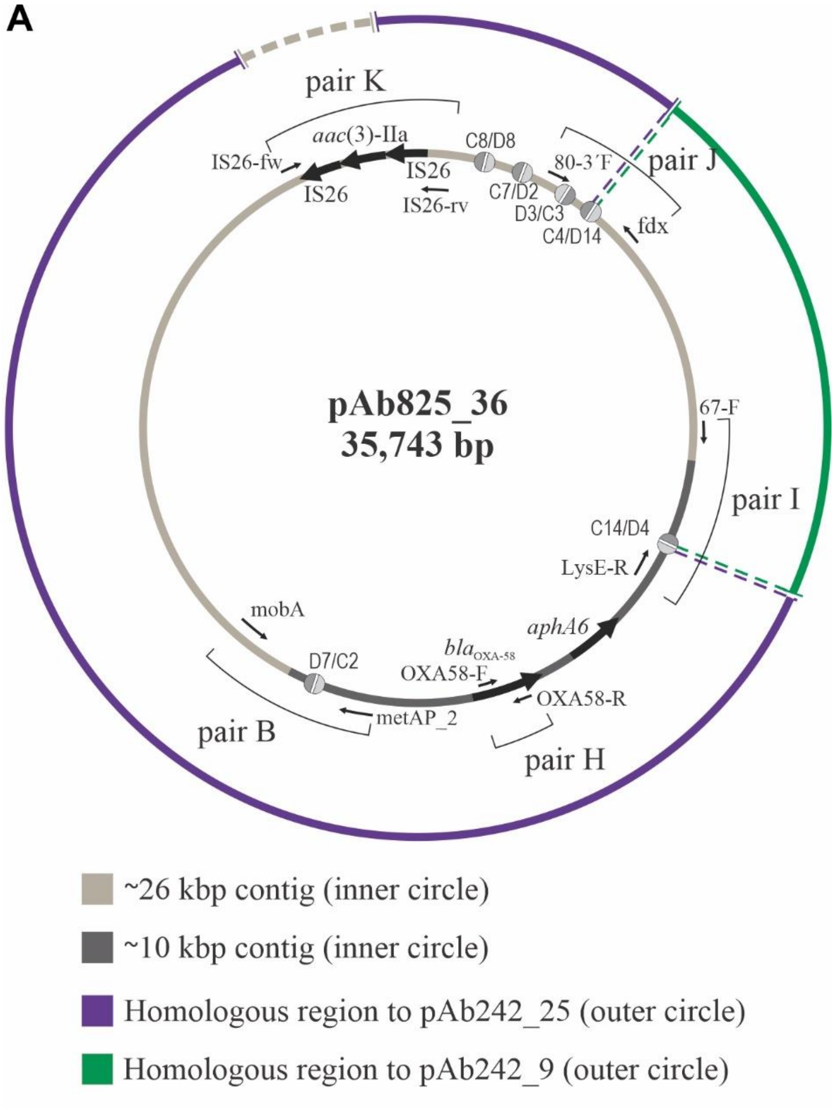

B

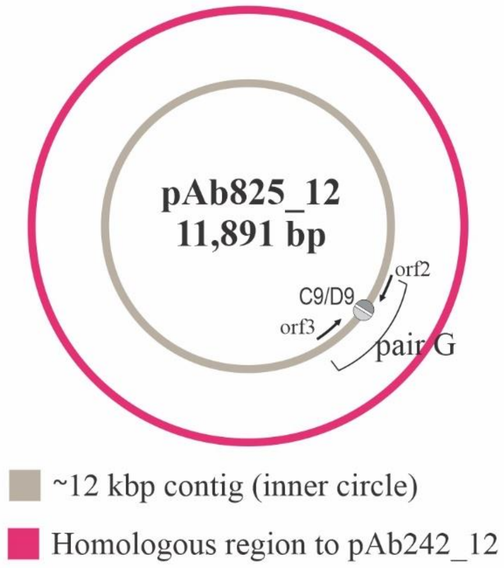

Legend to Figure S3. **Assembly of the two predominant plasmid forms present in the *A. baumannii* Ab825 strain from WGS data analysis complemented with gap closure strategies**.

The circular forms of pAb825_36 (**A**, inner circle) and pAb825_12 (**B**, inner circle) were obtained from three predominant plasmid contigs assembled from the pyrosequencing data. Two of these contigs could be merged by PCR using the primer sets I (detects C14/D4) and J (detects C4/D14) (Supplementary Table S1) into a circular structure of 35,743 bp designated pAb825_36 (A). The third contig was closed by PCR using the primer pair G (Supplementary Table 1) into a circular structure of 11,891 bp (B) identical to pAb242_12 (KY984046). This plasmid was designated pAb825_12. pAb825_36 and pAb825_12 were deposited in GenBank under accession number MG100202 and MG100203, respectively. In addition, primer pair K was used to confirm the presence in pAb825_36 of a composite transposon-like structure formed by two IS*26* elements bracketing an aminoglycoside resistance gene *aac(3)*-IIa. Relevant pXerC/D sites identified in these plasmids are indicated as ovals (not drawn to scale), with the XerC and XerD recognition sequences depicted as dark gray and light gray semi-ovals, respectively. For each final plasmid structure, the outer circles highlight the regions that display homology to Ab242 plasmids (Cameranesi et al., 2018). Thus, pAb825_36 (A) contains a region of complete identity to pAb242_9 (spanning from C14/D4 to C4/D14, highlighted in green in the outer circle) and extensive regions of identity to pAb242_25 (indicated in violet in the outer circle).

Supplementary Figure S4.

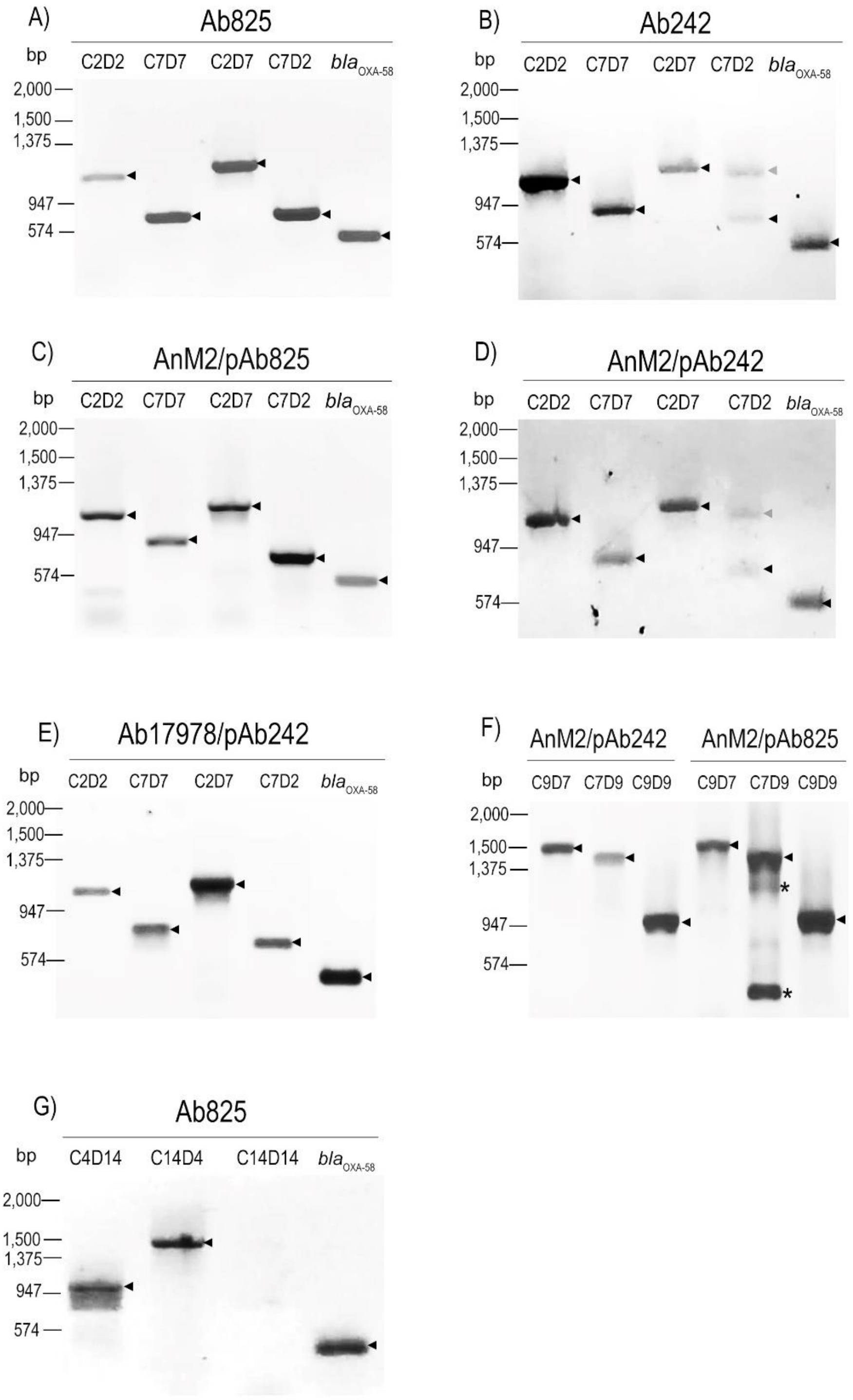

Legend to Figure S4. PCR evidence for the coexistence of different plasmid structural variants resulting from SSR mediated by active pairs of pXerC/D sites in clonal populations of the different *Acinetobacter* strains analyzed in this study.

The PCR reactions were conducted using different combinations of individual primers (detailed in Supplementary Table S1) and as templates plasmid mixtures extracted from clonal populations of the *A. baumannii* strains or the different *Acinetobacter* model strains transformed with plasmids extracted from these strains, as indicated above each figure. The individual pXerC/D sites identified in each case are indicated above the corresponding lanes. The amplified mixtures

were analyzed by agarose gel electrophoresis/ethidium bromide staining (the corresponding negative images are shown) followed by the cloning and sequencing of the amplified DNA bands to differentiate between bands carrying the indicated pXerC/D site (labeled with a closed arrowhead (◀) at the right of each lane) from nonspecific products (labeled with asterisks). The sizes of the expected amplification bands are also indicated in Supplementary Table S1. **A.** Coexistence of PSVs derived from intramolecular inversions due to SSR mediated by the sister pair C2/D2||C7/D7 and the resulting hybrid pair C2/D7||C7/D2 in clonal cultures of *A. baumannii* Ab825. The amplification band indicating the presence of the $bla_{\mathrm{OXA\text{-}58}}$ gene in the analyzed plasmid mixture is also shown (last lane at the right). **B.** Same, for *A. baumannii* Ab242. The upper band indicated with an open arrowhead is a different structural variant which will be described in a further work. **C.** Same, for *A. nosocomialis* M2 transformed with Ab825 plasmids (AnM2/pAb825). **D.** Same, for *A. nosocomialis* M2 transformed with Ab242 plasmids (AnM2/pAb242). **E.** Same, for *A. baumannii* ATCC17978 transformed with Ab242 plasmids (Ab17978/pAb242). **F.** Detection of co-integrates resulting from intermolecular fusions mediated by the sister pair C7/D7||C9/D9 in plasmid mixtures extracted from clonal cultures of AnM2/pAb825 and AnM2/pAb242 cells. The existence of PSVs in which the reverse (resolution) reaction regenerated the (original) C9/D9 site is also shown. The presence of the other member of the pair, C7/D7, in the same plasmid extracts is shown above (see **C** and **D)**. **G.** PCR assay for the presence of C4/D14, C14/D4, and C14/D14 sites in plasmid extracts of Ab825 clonal cultures.

Supplementary Figure S5.

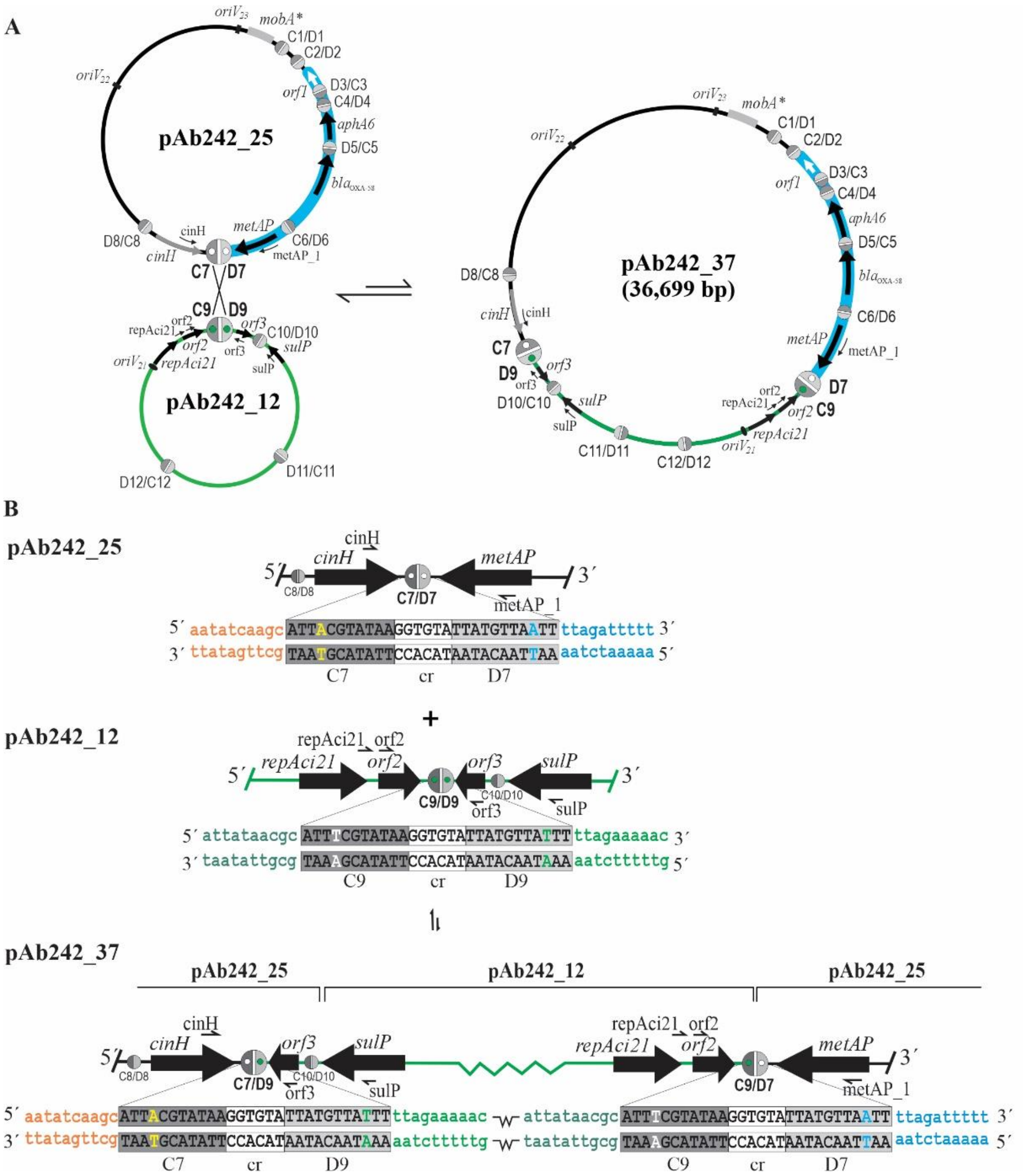

Legend to Figure S5. Intermolecular fusions and intramolecular resolutions resulting from site-specific recombination between sister pairs of pXerC/D sites in *Acinetobacter* plasmids.

A. Scheme depicting the formation of the pAb242_37 co-integrate by inter-molecular SSR mediated by the sister pair formed by the C7/D7 and C9/D9 sites located in the bi-replicon pAb242_25 and the single replicon pAb242_12, respectively. The reverse reaction conducting to the resolution of this co-integrate by intra-molecular SSR, now mediated by the directly-oriented (hybrid) pair C9/D7 and C7/D9, is also shown. The PCR primers used to identify the presence of specific pXerC/D sites are indicated. Plasmid pAb242_12 has been arbitrarily colored in green to facilitate visualization of the SSR. **B.** Enlarged vision of the SSR reactions conducing to the fusion and resolution events described above. Note that the cr spacer sequences are identical in all recombining sites, while the XerC- and XerD- half-sites show some nucleotide differences between sites. These variations have been highlighted in uppercase colored letters to facilitate visualization of the SSR events.

Table S1. Oligonucleotide primers used for PCR analysis of XerC/D sites in *Acinetobacter* plasmids in this work

Primer pair		Nucleotide sequence (5´→3´)	Amplification product size (bp)/ Comments	Source or reference
**A**	mobA	GTACTCCTGATAAAGTGCATCACGTTGTGC	1,110 bp Detects XerC/D site C2D2 (see GenBank accession number: ON060992)	This work
	orf1	GAACTAGTACCTGGCAACCATCATGA		
**B**	mobA	GTACTCCTGATAAAGTGCATCACGTTGTGC	1,233 bp Detects C2D7 (see GenBank accession number ON060990)	This work
	metAP_2	GCGTCGTTTAGTTGATACTACC		
**C**	orf1	GAACTAGTACCTGGCAACCATCATGA	604 bp Detects C7D2 (see GenBank accession number ON060991)	This work
	cinH	GACCAAGCTGAAGCTTTACGACAAGCATGG		Cameranesi et al., 2018
**D**	metAP_1	CGCTCAATAAATGCTCAGGCTGCTTTCTGG	1,642 bp Detects C9D7 (see GenBank accession number OM876186)	Cameranesi et al., 2018
	repAci21	AGCTACAAAATACAGTGTTAGTG		This work
**E**	metAP_2	GCGTCGTTTAGTTGATACTACC	727 bp Detects C7D7 (see GenBank accession number ON060993)	This work
	cinH	GACCAAGCTGAAGCTTTACGACAAGCATGG		Cameranesi et al., 2018
**F**	sulP	GCTGGCTGTGCGATGATTGG	1,522 bp Detects C7D9 (see GenBank accession number OM876185)	This work
	cinH	GACCAAGCTGAAGCTTTACGACAAGCATGG		Cameranesi et al., 2018
**G**	orf2	TGGAGCAAAACAGGCAAAAC	874 bp Detects C9D9 (see GenBank accession number ON060994)	This work
	orf3	CTCAGGGCTGAATCTGTCAC		This work
**H**	OXA-58F	AAGTATTGGGGCTTGTGCTG	386 bp Detects *bla*$_{OXA-58}$ gene	This work
	OXA-58R	TACGACGTGCCAATTCTTGA		Ravasi et al., 2011
**I**	67-Fw	AGCGTAAAAGACCAGTTTGGCGT	1,648 bp Detects C14D4	Cameranesi et al., 2018
	LysE-R	AAACTGTCGCACCTCATGTTTGA		This work
**J**	80-3`F	GTCATCAATGCACAGCGGT	967 bp Detects C4D14	Cameranesi et al., 2018
	fdx	ACACTGACGGCAAGCCTCAGC		This work
**K**	IS26-fw	ATCATGCGATAACTCACCATC	3,077 bp Detects Tn*aac*(3)II-a	This work
	IS26-rv	TGCTCGACAGAACTTACCC		

**L**	67-Fw	AGCGTAAAAGACCAGTTTGGCGT	1,984 bp<br>Detects<br>C14D14	Cameranesi et al., 2018
	fdx	ACACTGACGGCAAGCCTCAGC		This work
